# The association between community-level socioeconomic status and cognitive function among Chinese middle-aged and older adults: a study based on the China Health and Retirement Longitudinal Study (CHARLS)

**DOI:** 10.1186/s12877-022-02946-3

**Published:** 2022-03-22

**Authors:** Yan Liu, Zhaorui Liu, Richard Liang, Yanan Luo

**Affiliations:** 1 Institute of Population Research, Peking University, Beijing, China; 2 Peking University Sixth Hospital, Beijing, China; 3 School of Medicine, Stanford University, California, USA; 4 Department of Global Health, School of Public Health, Peking University, No.38 Xueyuan Road, Haidian District, Beijing, PR China 100191

**Keywords:** Cognitive function, Community environment, Community SES, Structural equation modeling

## Abstract

**Background:**

Although numerous studies focused on the relationship between area socioeconomic status (SES) and health, only a few of them investigated how community-level SES was linked to late-life cognitive function as well as the potential pathways underlying this association, and very few of them focused on the context of China. This study examined how community-level SES was linked to cognitive function and the potential pathways underlying this association among middle-aged and older adults in China.

**Methods:**

Data was drawn from the waves 1–4 of China Health and Retirement Longitudinal Study. We measured cognitive function with the components of the Telephone Interview of Cognitive Status battery. Community-level SES was derived from a sum of z scores of the percentage of the illiterate and the per-capita net income status within communities. We adopted two-level hierarchical linear regression models to explore the associations between community-level SES and cognitive function. A multilevel mediation analysis with structural equation modeling was undertaken to disaggregate the direct and indirect pathways of the associations.

**Results:**

Higher community-level SES was associated with better cognitive function (β = 0.562, 95% CI = 0.390, 0.734), and this significant association was only present in rural participants, not in urban participants. Furthermore, we discovered the mediating effects of outdoor exercise facilities within communities (β = 0.023, 95% CI = 0.000, 0.056) and individual-level SES (β = 0.108, 95% CI = 0.057, 0.156) to explain the relationship between community SES and cognitive function.

**Conclusions:**

These findings highlight the importance of community environmental interventions in maintaining individuals’ cognitive health in China, especially for older adults. Our results provided solid empirical evidence for reducing mental health inequalities in China, and suggested that developing an aging-friendly environment and properly distributing community resources are important to improve cognitive function of older adults.

**Supplementary Information:**

The online version contains supplementary material available at 10.1186/s12877-022-02946-3.

## Introduction

Cognitive impairment refers to deficits or abnormalities in attention, memory, language, learning new things or making sense, which affects a person’s daily life [1, 2]. Diseases associated with cognitive impairment, particularly dementia, lead to decreasing living standards and bring the huge burden of diseases [3, 4]. Dementia has become one of the leading causes of death around the world, especially for adults aged 70 years and above [5]. It was estimated that more than 43 million people suffered from dementia worldwide in 2016, of which one quarter was from China [5]. The prevalence of dementia among older adults in China was around 6% in 2018 [6], and Alzheimer’s disease was one of the 17 leading causes of disability-adjusted life-years (DALYs) in 2019 [7]. Thus, in this context, the prevention of cognitive impairment in China is clearly important.

Following the socio-ecological theory, community-level socioeconomic status (SES) is the key context for determining individuals’ health [8]. Previous studies found that people residing in lower SES communities had worse cognitive function than those in more affluent communities [9–13]. Compared with younger adults, older adults’ health conditions are more vulnerable to community-level SES. With age-related health declining and social space shrinking, older adults are more likely to spend time communities inside and tend to engage in more light activity compared with the younger [9, 14–16]. Although numerous studies focused on the relationship between area SES and health [9, 17–25], only a few of them investigated how community-level SES was linked to late-life cognitive function [9, 11], and very few of them focused on the context of China. To our knowledge, there has been only one study examining the association between community-level SES and cognitive health in mainland China; however, it failed to explore the potential pathways underlying this association [17]. With the rapid and unbalanced economic development in China, considerable health disparities at the area level are imposed [26]. Besides, the disparities of socioeconomic conditions and health care services’ supply and access between urban and rural contribute to the health inequalities among urban and rural population in China [26–28]. It is essential to identify whether community SES is related to late-life cognitive function and whether there is an urban-rural difference in this relationship, which is helpful for formulating the area-level mental health interventions policies, narrowing the urban-rural gap in mental health inequalities, and achieving the goal of healthy ageing in China.

Two major pathways linking community-level SES to individual health have been proposed. The first pathway suggests that the impact of community-level SES on health is independent of individual-level SES, which was through the accessibility of material and social resources and the improvement of physical and social environments of communities [10, 29–31]. Individuals in advantaged community environments were given access to more health resources, such as healthcare amenities, social engagement facilities and outdoor exercise facilities [9, 13, 30, 32, 33]. The second one argues that the health consequence of community level SES was through the pathway of individual level SES [29]. Individual-level SES may contain information on a person’s ability to obtain resources and process information, which affects individuals’ health [12].

Using nationally representative longitudinal survey data, this study explored how community-level SES was linked to cognitive function as well as the potential pathways of this association among middle-aged and older adults in China. Moreover, given the dramatic urban-rural differences in China, this study also examined whether the urban-rural differences existed in the association between community SES and cognitive function. Our study contributes to the existing literature on the mechanisms underlying the relationship between community-level SES and cognitive function in the context of developing countries.

## Methods

### Data

The data in our study was drawn from the China Health and Retirement Longitudinal Study (CHARLS). CHARLS used a multistage, stratified, probability proportional to size (PPS) sampling method, covering 28 provinces, 150 counties and 450 communities in China, which ensured the national representativeness of the sample [34]. Respondents aged 45 years old and above were selected. CHARLS collected detailed information on individual characteristics (such as demographics characteristics, health status and socioeconomic position) and community characteristics (such as the population and occupation status of communities, health facilities and socioeconomic status) by face-to-face computer-aided personal interviews (CAPI). The baseline survey of CHARLS was conducted in 2011, following up every 2 years from 2013 to 2018. The response rates of all samples were higher than 80% in each wave [35].

**Fig. 1:**
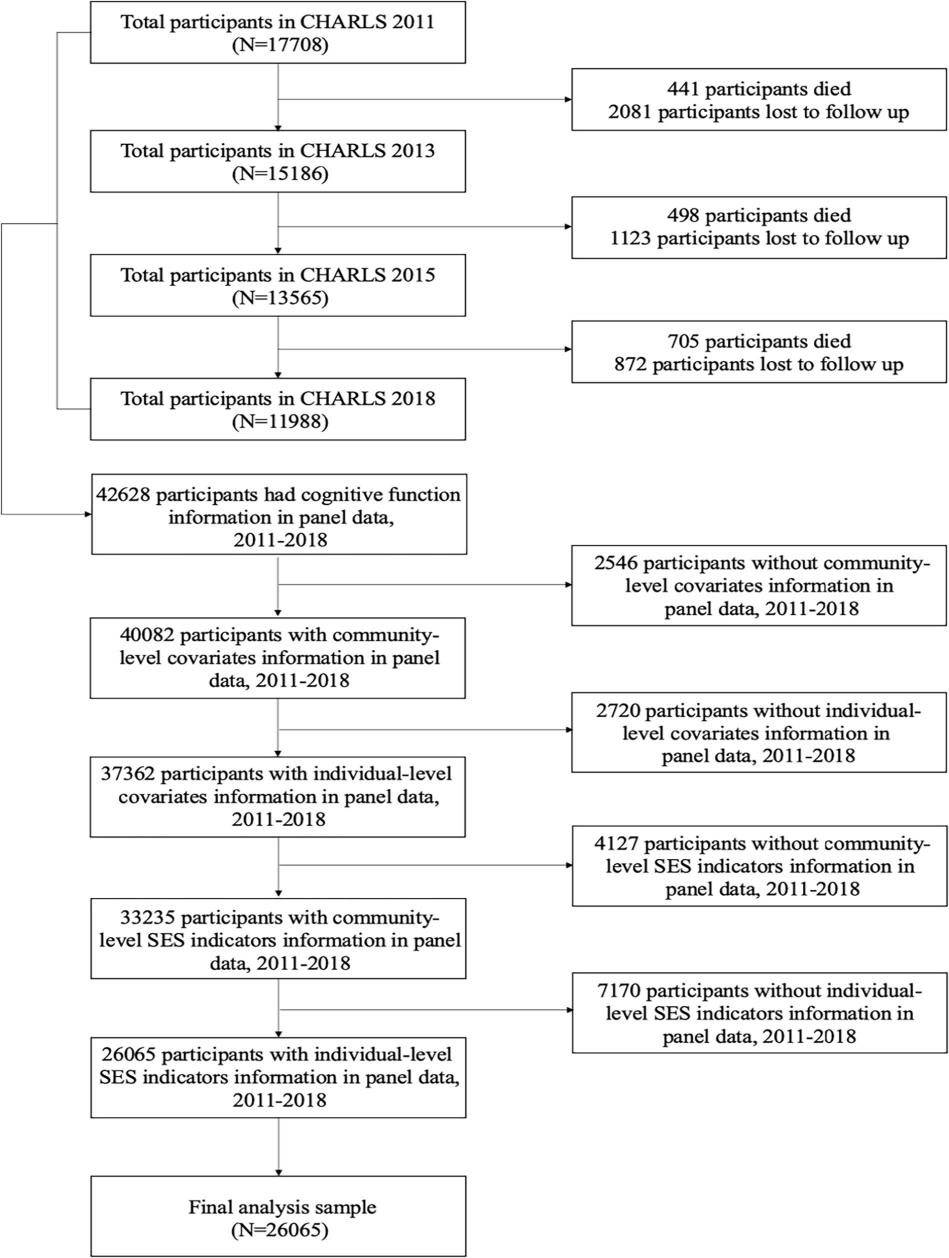
Flow chart of sampling of this study. Note. *CHARLS* China Health and Retirement Longitudinal Survey

This study utilized four waves of CHARLS (2011–2018) in total. Of 17,708 respondents in CHARLS 2011, 441 died and 2081 were lost to follow up, leaving 15,186 participants re-interviewed in CHARLS 2013. Among those people, 498 died and 1123 were lost to follow up, with 13,565 respondents re-interviewed in CHARLS 2015. Of these, except for 705 who died and 872 lost to follow up, 11,988 participants were re-interviewed in CHARLS 2018. Individuals who did not participate in the follow-ups from 2013 to 2018 were excluded, leaving 42,628 cases with cognitive function information in this study. Then, participants without community-level covariates (2546) and individual-level covariates (2720) information, and cases with missing community-level SES indicators (4127) and individual-level SES indicators (7170) were excluded from waves 2011–2018. Finally, we restricted our finalized analysis to 26,065 individuals aged 45 years or older (Fig. 1).

### Cognitive function

The dependent variable in this study was cognitive function, which represented the abilities of memory, orientation, mental intactness and attention [36]. The items used to measure cognitive function in CHARLS were the components of the Telephone Interview of Cognitive Status battery (TICS) [35]. Memory was measured by an immediate word recall test (0 to 10 scores) and a delayed recall test (0 to 10 scores), asking respondents to repeat, regardless of the order, 10 Chinese nouns immediately and 5 min later. CHARLS examined orientation through the respondents’ recognition of date (month, day, year), season, and the day of the week (0 to 5 scores). Visuoconstruction was assessed by asking individuals to exactly redraw a picture of overlapping pentagons on the paper (0 to 1 score). Serial subtraction of 7 s from 100 was used to measure numeric ability (0 to 5 scores). The total cognitive function score varied from 0 to 31, with higher values meaning better cognitive function [30]. In addition, this study also assessed two key elements of cognitive function: mental intactness and episodic memory. Mental intactness was formed by numeric ability, orientation and visuoconstruction, with a range of 0 to 11. Episodic memory was calculated by the average of immediate and delayed word recall scores, which ranged from 0 to 10 [37].

### Community-level SES

Based on the community questionnaire of CHARLS, the percentage of the illiterate and the per-capita net income status in the previous year within communities were used together to measure community-level SES in the current study. We first computed z scores for education and income variables, and then summed them into an overall index representing the community SES with a mean of zero and SD of 1. The full range of community-level SES was from − 4.79 to 6.91, with higher scores suggesting a higher level of community SES.

### Mediators

According to Robbert’s theoretical framework on the community SES and health [29], both individual-level and community-level variables were selected as mediators. At the individual level, individual SES was selected as the mediator, with a mean of zero and SD of 1 and ranging from − 17.59 to 43.70, which was calculated by the sum of z scores of the years of schooling and per household income. Higher values denoted higher individual SES.

At the community level, both physical and social environments factors were chosen to be the mediators. Six indicators of community physical environment were used to capture healthcare service and outdoor space, and seven indicators of social environment were selected to capture the social organizations and public amenities in the community.

As to physical environments, we measured the healthcare service on the availability of general hospitals, specialized hospitals, Chinese medicine hospitals, community health care centers, and community health care medical posts in the community (yes = 1). Outdoor space was measured by the degree of handicapped access for community dwellers (ranging from *no handicapped access* = 1 to *very convenient* = 7). For the social environments, we measured the variety of social organizations by counting the availability of four types of voluntary associations, including calligraphy and painting, dancing or other exercises, helping older people and the handicapped and the elderly association (ranging from 0 to 4). Public amenities were assessed by whether the community has outdoor exercise facilities (yes = 1), whether the community has rooms for card games and chess games (yes = 1), and the number of libraries (0 to 4).

### Covariates

Age, sex (male or female), residence (urban or rural), occupation (agricultural work or non-agricultural work), marital status (unmarried or married), and activities of daily living (ADLs) (unimpaired or impaired), and the percentage of residents with non-agricultural work at the community level were selected as covariates.

### Statistical analysis

This study adopted two-level hierarchical linear regression models (HLM) to explore the associations between community-level SES and cognitive function. A multilevel mediation analysis with structural equation modeling (MMSEM) was undertaken to disaggregate the direct and indirect pathways of the associations. Bayesian estimation was applied to perform a test of indirect and total effects from the model. All of the analyses were carried out by using Mplus Version 8.3 [38].

## Results

### Characteristics of participants

Table 1 presents the characteristics of participants (Table 1). Among all respondents, the average score of cognitive function was 14.86 (±5.46). The mean age of participants was 60.21 (±9.05). 50.25% of all samples were male. Almost half (49.42%) of the respondents had agricultural work. Most of the participants were married (87.53%) and had unimpaired ADLs (82.32%). Years of schooling and per household income of the whole sample averaged at 5.14 and 8910.97 Yuan, respectively.

**Table 1:** Characteristics of participants

Characteristics	Total (*n* = 26,065)	Urban (*n* = 9155)	Rural (*n* = 16,910)	*P* value for urban-rural difference
	N (%) or Mean (SD)	N (%) or Mean (SD)	N (%) or Mean (SD)	
**Outcome**				
Cognitive function	14.86 (5.46)	16.05 (5.27)	14.22 (5.45)	< 0.001
**Community-level SES variables**				
Percentage of the illiterate (%)	11.90 (12.69)	9.11 (9.97)	13.41 (13.72)	< 0.001
Per-capita net income, Yuan	4895.66 (5751.73)	6802.77 (7306.49)	3863.15 (4365.55)	< 0.001
Community-level SES	0.00 (1.00)	0.38 (1.03)	−0.20 (0.92)	< 0.001
*Community-level SES, n (%)* ^a^				< 0.001
Low	8685 (33.32)	1697 (18.54)	6988 (41.32)	
Middle	8683 (33.31)	2741 (29.94)	5942 (35.14)	
High	8697 (33.37)	4717 (51.52)	3980 (23.54)	
**Individual-level SES variables**				
Years of schooling, years	5.14 (4.56)	6.37 (4.74)	4.49 (4.32)	< 0.001
Per household income, Yuan	8910.97 (20,379.19)	13,830.77 (28,937.33)	6247.42 (12,909.19)	< 0.001
Individual-level SES	−0.00 (1.00)	0.33 (1.24)	−0.18 (0.78)	< 0.001
*Individual-level SES, n (%)* ^a^				< 0.001
Low	8685 (33.32)	2027 (22.14)	6658 (39.37)	
Middle	8691 (33.34)	2545 (27.80)	6146 (36.35)	
High	8689 (33.34)	4583 (50.06)	4106 (24.28)	
**Community-level sociodemographic variables**				
Percentage of residents with non-agricultural work (%)	52.39 (24.64)	74.42 (22.58)	40.46 (15.96)	< 0.001
Health care facilities, *n* (%)				< 0.001
No	24,529 (94.11)	7964 (86.99)	16,565 (97.96)	
Yes	1536 (5.89)	1191 (13.01)	345 (2.04)	
Handicapped access	1.91 (1.43)	2.52 (1.68)	1.58 (1.14)	< 0.001
Outdoor exercise facilities, *n* (%)				< 0.001
No	17,972 (68.95)	4183 (45.69)	13,789 (81.54)	
Yes	8093 (31.05)	4972 (54.31)	3121 (18.46)	
Voluntary social organizations	0.97 (1.15)	1.68 (1.28)	0.59 (0.86)	< 0.001
Libraries	0.58 (0.61)	0.74 (0.63)	0.49 (0.58)	< 0.001
Rooms for card games and chess games, *n* (%)				< 0.001
No	17,316 (66.43)	3961 (43.27)	13,355 (78.98)	
Yes	8749 (33.57)	5194 (56.73)	3555 (21.02)	

*SES* socioeconomic status

^a^Variable was treated as a continuous variable but is presented categorically for descriptive purposes

At the community level, the average percentage of the illiterate in communities was 11.90, and the per-capita net income was 4895.66 Yuan. 24.64% of residents were engaged in non-agricultural work. Only 5.89% of residents lived in communities with health care facilities, and over 68% were in communities without outdoor exercise facilities. On average, participants lived in communities with a low level of handicapped access and the average score was 1.91 (±1.43). Moreover, the score of the variety of voluntary social organizations and the number of libraries was 0.97 (±1.15) and 0.58 (±0.61), respectively.

In comparison to urban individuals, rural individuals tended to live in lower SES communities and have worse cognitive function. Also, rural respondents were more likely to be male, married, and had agricultural work and impaired ADLs. More details could be found in Table 1. More details could be seen in Supplemental Table 1.

### Multilevel regressions results of the association between community-level SES and cognitive function

Table 2 reports the results of multilevel linear regressions (Table 2). After controlling for age, sex, residence, occupation, marital status, ADLs and the percentage of residents with non-agricultural work, higher community-level SES was found to be associated with better cognitive function (β = 0.562, 95% CI = 0.390, 0.734). It was noteworthy that this significant association was only present in rural participants (β = 0.658, 95% CI = 0.416, 0.901), not in urban participants (β = 0.146, 95% CI = − 0.113, 0.405).

**Table 2:** Multilevel linear regressions of the association between community-level SES and cognitive function

Characteristics	Total (*n* = 26,065)	Urban (*n* = 9155)	Rural (*n* = 16,910)
Community-level SES	**0.562 (0.390, 0.734)**	0.146 (−0.113, 0.405)	**0.658 (0.416, 0.901)**
Percentage of residents with non-agricultural work	**2.488 (1.859, 3.117)**	**1.916 (0.887, 2.944)**	0.679 (−0.156, 1.513)
Age	**−0.153 (− 0.160, − 0.146)**	**−0.151 (− 0.162, − 0.140)**	**−0.157 (− 0.166, − 0.147)**
Sex (ref = male)			
Female	**−1.647 (−1.766, −1.528)**	**−0.910 (−1.105, − 0.716)**	**−2.066 (−2.215, −1.917)**
Residence (ref = urban)			
Rural	**0.491 (0.187, 0.795)**		
Occupation (ref = agricultural work)			
Non-agricultural work	**0.256 (0.114, 0.397)**	**0.787 (0.513, 1.061)**	0.029 (−0.136, 0.194)
Marital status (ref = unmarried)			
Married	**1.156 (0.965, 1.346)**	**1.089 (0.787, 1.392)**	**1.252 (1.010, 1.494)**
ADLs (ref = no-impaired)			
Impaired	**−1.059 (−1.221, −0.896)**	**− 1.606 (− 1.888, − 1.323)**	**−0.771 (− 0.968, − 0.575)**
ICC	0.103	0.131	0.118

*SES* socioeconomic status, *ADLs* activities of daily living, *ICC* interclass correlation coefficient

### MMSEM results of the total, direct and indirect effects of the pathways between community-level SES and cognitive function

The mediation analysis results of community-level SES and cognitive function can be seen in Table 3. Community-level SES had total (β = 0.380, 95% CI = 0.179, 0.570), direct effects (β = 0.265, 95% CI = 0.100, 0.430) and indirect effects (β = 0.111, 95% CI = 0.036, 0.184) on cognitive function. As for specific indirect effects, handicapped access (β = 0.000, 95% CI = − 0.009, 0.009), health care facilities (β = 0.000, 95% CI = − 0.015, 0.009), voluntary social organizations (β = 0.001, 95% CI = − 0.021, 0.025), libraries (β = − 0.002, 95% CI = − 0.022, 0.009) and rooms for card games and chess games within communities (β = − 0.014, 95% CI = − 0.059, 0.006) had no significant mediating effects on the association between community-level SES and cognitive function. The indirect pathways were dominantly through individual-level SES (β = 0.108, 95% CI = 0.057, 0.156) and outdoor exercise facilities (β = 0.023, 95% CI = 0.000, 0.056).

**Table 3:** Direct, indirect, and total effects of the pathways between community-level SES and cognitive function (*N* = 26,065)

Effects	Estimate (95% CI)
Total effects	**0.380 (0.179, 0.570)**
Direct effects	**0.265 (0.100, 0.430)**
Total indirect effects	**0.111 (0.036, 0.184)**
Specific indirect effects	
Community-level SES → Individual-level SES → Cognitive function	**0.108 (0.057, 0.156)**
Community-level SES → Handicapped access → Cognitive function	0.000 (− 0.009, 0.009)
Community-level SES → Health care facilities → Cognitive function	0.000 (− 0.015, 0.009)
Community-level SES → Outdoor exercise facilities → Cognitive function	**0.023 (0.000, 0.056)**
Community-level SES → Voluntary social organizations → Cognitive function	0.001 (− 0.021, 0.025)
Community-level SES → Libraries → Cognitive function	−0.002 (− 0.022, 0.009)
Community-level SES → Rooms for card games and chess games → Cognitive function	−0.014 (− 0.059, 0.006)

Multilevel structural equation models adjusted for the percentage of residents with non-agricultural work at the community level, age, sex, residence, occupation, marital status, ADLs at the individual level. The estimates are omitted for simplicity

*SES* Socioeconomic status, *CI* Confidence interval

**Fig. 2:**
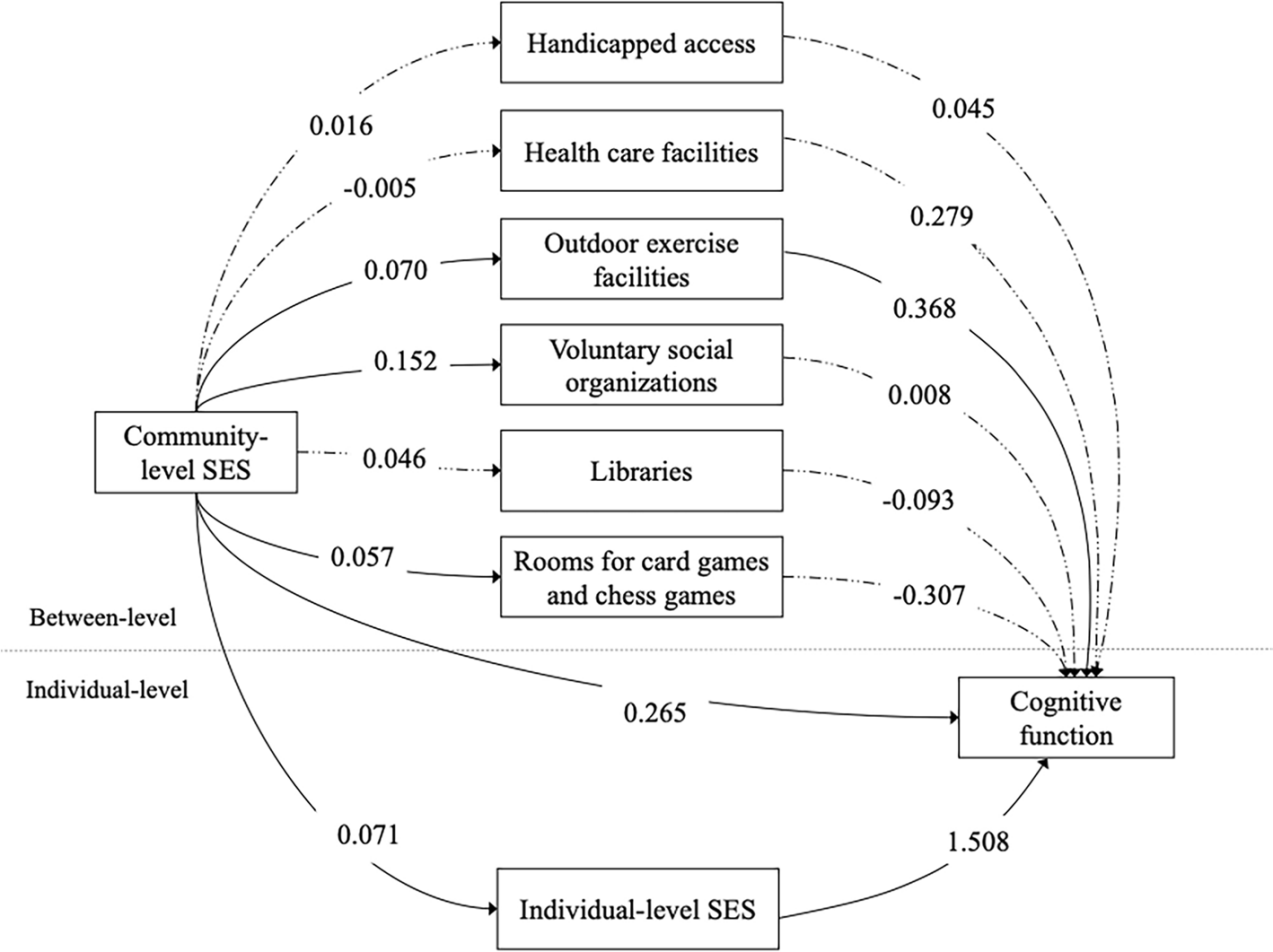
Pathways between community-level SES, mediators, and cognitive function (*N* = 26,065). Note. Multilevel structural equation models adjusted for the percentage of residents with nonagricultural work at the community level, age, sex, residence, occupation, marital status, and ADLs at the individual level. The estimates are omitted for simplicity. Significant pathways (solid lines) and insignificant pathways (dashed lines) between community-level SES, mediators, and cognitive function are presented here. *SES* socioeconomic status

Figure 2 shows the results of the MMSEM analysis. Outdoor exercise facilities and individual-level SES mediated the association between community-level SES and cognitive function. Residing in communities with outdoor exercise facilities was associated with higher cognitive scores (β = 0.368, 95% CI = 0.003, 0.660), and higher SES individuals were more prone to have better cognitive function (β = 1.508, 95% CI = 1.433, 1.573). Additionally, respondents who lived in higher SES communities were linked to be in higher individual-level SES (β = 0.071, 95% CI = 0.037, 0.103) and have better cognitive function (β = 0.265, 95% CI = 0.100, 0.430). Although higher SES communities were more likely to have rooms of card games and chess games (β = 0.057, 95% CI = 0.009, 0.099) and more types of voluntary social organizations (β = 0.152, 95% CI = 0.056, 0.227), having rooms of card games and chess games and more types of voluntary social organizations were not significantly associated with cognitive function.

## Discussion

Based on CHARLS longitudinal data from a nationally representative sample of Chinese middle-aged and older adults, we demonstrated a significant association between community-level SES and cognitive function as well as its urban-rural difference. Furthermore, we found the mediating effects of outdoor exercise facilities and individual-level SES to account for the relationship between community SES and cognitive function. To our best knowledge, this is the first study to detect the potential pathways underlying the linkage of community-level SES and cognitive function in mainland China.

We found that community-level SES was positively associated with cognitive function, and individuals residing in higher SES communities experienced increasing cognitive function. This finding is consistent with recent evidence from Singapore [39], the Netherlands [9], the United States [13] and India [40]. We also found the similar results from multilevel regression models at mental intactness and episodic memory aspects (see Supplemental Table 2). Besides, we observed that the relationship between community-level SES and cognitive function was significant in rural participants, but not in urban participants. These findings may provide support for the resource substitution theory, proposing that when resources substitute for each other, the presence of one makes the lack of one less detrimental. In contrast, the scarcer one resource is, the more valuable another becomes. That is, persons in disadvantaged status gain more health benefits than their advantaged counterparts [41]. People, in rural communities, generally have fewer basic infrastructure, housing, education and other socioeconomic resources than the urban residents [30, 42, 43]. And rural residents may gain more benefit from higher SES communities than urban residents.

Our findings showed that outdoor exercise facilities mediated the association of community SES and cognitive impairment. Higher SES communities are more likely to have easily accessible outdoor exercise facilities and other healthy living environments, which foster physical, mental and social wellbeing [17]. And residents living in environments with outdoor exercise facilities may have more opportunities to be engaged in physical exercises, social participation and social interactions [9, 17, 32], and further bring cognitive stimulation and improve cognitive function [44, 45]. This finding is in line with previous studies [11, 13] and may support the collective resources model, which argued that individuals in more affluent areas may benefit from area-level resources, over and above individual-level SES [46].

In addition, individual-level SES was found to be the potential pathway for the relationship between community-level SES and cognitive function. Two possible reasons may explain this mediating effect of individual-level SES. First, higher SES communities gather a large number of people with better socioeconomic conditions [9, 47], and these high SES individuals gain more health knowledge and find more ways to access psychological, social and behavioral resources which are conducive to their cognitive function than their low SES counterparts [2, 12, 25, 46, 48]. Second, higher SES individuals, particularly those who are well educated, have a greater capacity for cognitive reserve than people with lower SES [17, 49]. This result may give empirical evidence to support the cognitive reserve hypothesis, indicating that people with higher cognitive reserve could better cope with brain damage and cognitive aging as compared to their counterparts with lower cognitive reserve through neural reserve or neural compensation [13, 17, 49].

Moreover, this study found that the occupation type and marital status were two important risk factors for cognitive function, which are consistent with previous studies [50, 51]. The differences in health behaviors, physical activities and nutrition conditions as well as performance on cognitive function tests may explain the protective role of non-agricultural work in cognitive scores [50, 52]. As for the role of marital status, those married individuals may receive more social support, leading to fewer mental disorders [53]. Besides, in line with previous studies [51, 54], we found that females, older adults and those with disability were more likely to have poor cognitive function. Screening assessment should be conducted to those at high poor cognitive function risk, which may help to lower the chance of developing mild cognitive impairment.

### Limitations

Notably, there are several important limitations of the current study. First, we are only able to rule out associations between community-level SES and cognitive function rather than causations, although multiple years of CHARLS data have been combined to increase statistical power in this study. Second, owing to the unavoidable missing data in this large nationwide longitudinal survey, selection bias may be created by eliminating cases without complete information in the analysis phase. For example, it is reasonable to assume that individuals who died in the follow-ups from 2013 to 2018 tended to be frailer and cognitively impaired. Therefore, excluding these individuals from the analysis may have resulted in the sample variation in cognitive function, thereby leading to an underestimation of cognitive impairment associated with lower community-level SES. Third, due to the limitations of data, we could not involve more confounding factors in this study, such as the duration of exposure to community environments, the number of chronic diseases, general and central obesity, chronic diseases CVD and self-reported health status. Accordingly, the results of this study should be interpreted with caution. Fourth, a comprehensive look at community-level SES is needed in the future, given that we only introduced education and income as measures of community-level SES in the present study.

## Conclusion

These findings highlight the importance of improved socioeconomic context of community in maintaining individuals’ cognitive health in China, especially for older adults who are more susceptible to community characteristics. Our results provided solid empirical evidence for reducing mental health inequalities between communities with different SES levels and between urban and rural areas in China, and suggested that community environmental interventions including developing an aging-friendly environment and properly distributing community resources (such as providing outdoor exercise facilities and other cognitively stimulating amenities) could be implemented to improve the cognitive function of older adults. And these community environmental prevention interventions should implement according to the risk profiles of target population, such as different levels of individual SES.

## Supplementary Information

**Additional file 1: Supplemental Table 1.** Characteristics of participants. **Supplemental Table 2.** Multilevel linear regressions of the association between community-level SES and mental intactness and episodic memory.

## Data Availability

The dataset supporting the conclusions of this article is available at http://charls.pku.edu.cn/en.

## Abbreviations

SES: Socioeconomic status
DALYs: Disability-adjusted life-years
CHARLS: China Health and Retirement Longitudinal Study
PPS: Probability proportional to size
CAPI: Computer-aided personal interviews
TICS: Telephone Interview of Cognitive Status battery
ADLs: Activities of daily living
HLM: Hierarchical linear regression models
MMSEM: Multilevel mediation analysis with structural equation modeling
ICC: Interclass correlation coefficient
CI: Confidence interval
