# Supplementary material for: The association between community-level socioeconomic status and cognitive function among Chinese middle-aged and older adults: a study based on the China Health and Retirement Longitudinal Study (CHARLS)
Source: BMC Geriatr. 2022 Mar 22;22:239. doi: 10.1186/s12877-022-02946-3 (PMC8941774; doi:10.1186/s12877-022-02946-3)
Supplement: Supplementary file 1 — Additional file 1: Supplemental Table 1. Characteristics of participants. Supplemental Table 2. Multilevel linear regressions of the association between community-level SES and mental intactness and episodic memory. [file 12877_2022_2946_MOESM1_ESM.docx]

**Supplemental Table 1** Characteristics of participants

| Characteristics | Total (n=26065) |  | Urban (n=9155) |  | Rural (n=16910) |
| --- | --- | --- | --- | --- | --- |
|  | N (%) or Mean (SD) |  | N (%) or Mean (SD) |  | N (%) or Mean (SD) |
| **Outcome** |  |  |  |  |  |
| Cognitive function | 14.86 (5.46) |  | 16.05 (5.27) |  | 14.22 (5.45) |
| **Community-level SES variables** |  |  |  |  |  |
| Percentage of the illiterate (%) | 11.90 (12.69) |  | 9.11 (9.97) |  | 13.41 (13.72) |
| Per capita net income, Yuan | 4895.66 (5751.73) |  | 6802.77 (7306.49) |  | 3863.15 (4365.55) |
| Community-level SES | 0.00 (1.00) |  | 0.38 (1.03) |  | -0.20 (0.92) |
| *Community-level SES, n (%) ** |  |  |  |  |  |
| Low | 8685 (33.32) |  | 1697 (18.54) |  | 6988 (41.32) |
| Middle | 8683 (33.31) |  | 2741 (29.94) |  | 5942 (35.14) |
| High | 8697 (33.37) |  | 4717 (51.52) |  | 3980 (23.54) |
| **Individual-level SES variables** |  |  |  |  |  |
| Years of schooling, years | 5.14 (4.56) |  | 6.37 (4.74) |  | 4.49 (4.32) |
| Per household income, Yuan | 8910.97 (20379.19) |  | 13830.77 (28937.33) |  | 6247.42 (12909.19) |
| Individual-level SES | -0.00 (1.00) |  | 0.33 (1.24) |  | -0.18 (0.78) |
| *Individual-level SES, n (%) ** |  |  |  |  |  |
| Low | 8685 (33.32) |  | 2027 (22.14) |  | 6658 (39.37) |
| Middle | 8691 (33.34) |  | 2545 (27.80) |  | 6146 (36.35) |
| High | 8689 (33.34) |  | 4583 (50.06) |  | 4106 (24.28) |
| **Community****-level sociodemographic variables** |  |  |  |  |  |
| Percentage of residents with nonagricultural work (%) | 52.39 (24.64) |  | 74.42 (22.58) |  | 40.46 (15.96) |
| Health care facilities, n (%) |  |  |  |  |  |
| No | 24529 (94.11) |  | 7964 (86.99) |  | 16565 (97.96) |
| Yes | 1536 (5.89) |  | 1191 (13.01) |  | 345 (2.04) |
| Handicapped access | 1.91 (1.43) |  | 2.52 (1.68) |  | 1.58 (1.14) |
| Outdoor exercise facilities, n (%) |  |  |  |  |  |
| No | 24529 (94.11) |  | 7964 (86.99) |  | 16565 (97.96) |
| Yes | 1536 (5.89) |  | 1191 (13.01) |  | 345 (2.04) |
| Voluntary social organizations | 0.97 (1.15) |  | 1.68 (1.28) |  | 0.59 (0.86) |
| Libraries | 0.58 (0.61) |  | 0.74 (0.63) |  | 0.49 (0.58) |
| Rooms for card games and chess games, n (%) |  |  |  |  |  |
| No | 17316 (66.43) |  | 3961 (43.27) |  | 13.355 (78.98) |
| Yes | 8749 (33.57) |  | 5194 (56.73) |  | 3555 (21.02) |
| **Individual-level sociodemographic variables** |  |  |  |  |  |
| Age, years | 60.21 (9.05) |  | 60.47 (9.36) |  | 60.06 (8.87) |
| Sex, n (%) |  |  |  |  |  |
| Male | 13098 (50.25) |  | 4381 (47.85) |  | 8717 (51.55) |
| Female | 12967 (49.75) |  | 4774 (52.15) |  | 8193 (48.45) |
| Occupation, n (%) |  |  |  |  |  |
| Agricultural work | 12882 (49.42) |  | 2196 (22.90) |  | 10686 (63.19) |
| Nonagricultural work | 13183 (50.58) |  | 6959 (76.01) |  | 6224 (36.81) |
| Marital status, n (%) |  |  |  |  |  |
| Unmarried | 3250 (12.47) |  | 1228 (13.41) |  | 2022 (11.96) |
| Married | 22815 (87.53) |  | 7927 (86.59) |  | 14888 (88.04) |
| ADLs, n (%) |  |  |  |  |  |
| No-impaired | 21457 (82.32) |  | 7836 (85.59) |  | 13621 (80.55) |
| Impaired | 4608 (17.68) |  | 1319 (14.41) |  | 3289 (19.45) |

*Note:* SES = socioeconomic status; ADLs = activities of daily living.

*Variable was treated as a continuous variable but is presented categorically for descriptive purposes.

**Supplemental Table 2.** Multilevel linear regressions of the association between community-level SES and mental intactness and episodic memory (N=26065)

| Characteristics | Mental intactness |  | Episodic memory |
| --- | --- | --- | --- |
| Community-level SES | **0.305 (0.229, 0.382)** |  | **0.101 (0.047, 0.156)** |
| Percentage of residents with non-agricultural work | **1.422 (1.111, 1.732)** |  | **0.724 (0.513, 0.934)** |
| Age | **-0.057 (-0.061, -0.053)** |  | **-0.048 (-0.050, -0.045)** |
| Sex (ref = male) |  |  |  |
| Female | **-1.373 (-1.436, -1.310)** |  | **-0.135 (-0.176, -0.093)** |
| Residence (ref = urban) |  |  |  |
| Rural | **-0.218 (-0.369, -0.068)** |  | **0.283 (0.181, 0.385)** |
| Occupation (ref = agricultural work) | |  |  |
| Non-agricultural work | **0.159 (0.084, 0.234)** |  | **0.050 (0.001, 0.099)** |
| Marital status (ref = unmarried) | |  |  |
| Married | **0.512(0.411, 0.613)** |  | **0.322 (0.255, 0.388)** |
| ADLs (ref = no-impaired) |  |  |  |
| Impaired | **-0.616 (-0.702, -0.530)** |  | **-0.244 (-0.280, -0.167)** |
| ICC | 0.068 |  | 0.082 |

*Note:* SES = socioeconomic status; ADLs = activities of daily living.
